# Studying polymorphic variants of the *NAT2* gene (NAT2*5 and NAT2*7) in Nenets populations of Northern Siberia

**DOI:** 10.1186/s12863-020-00909-4

**Published:** 2020-10-22

**Authors:** Roza Pavlovna Tiis, Ludmila Pavlovna Osipova, Daria Veniaminovna Lichman, Elena Nikolaevna Voronina, Maxim Leonidovich Filipenko

**Affiliations:** 1 Institute of Cytology and Genetics SB RAS, 630090 Novosibirsk, Russia; 2 Novosibirsk State University, Russian Federation, 630090 Novosibirsk, Russia; 3 Institute of Chemical Biology and Fundamental Medicine SB RAS, Novosibirsk, Russia

**Keywords:** Xenobiotics, *NAT2* gene, NAT2*5, rs1801280, NAT2*7, rs1799931, Forest and Tundra Nenets, Northern Siberia

## Abstract

**Background:**

N-acetyltransferase 2 plays a crucial role in the metabolism of a wide range of xenobiotics, including many drugs, carcinogens, and other chemicals in the human environment. The article presents for the first time data on the frequency of two important “slow” variants of *NAT2* gene (NAT2*5, rs1801280 and NAT2*7, rs1799931*),* which significantly affect the rate of xenobiotics acetylation, among representatives of indigenous populations of Forest and Tundra Nenets in Northern Siberia. The aim of this study was to identify the frequencies of these variants and compare them with frequencies in other ethnic populations.

**Results:**

NAT2*5 (*T341C*) genotyping revealed frequencies of 28,0% and 38,6% for Tundra and Forest Nenets, respectively. The frequencies of NAT2*7 (*G857A*) variant were 9,8% and 8,2% for Tundra and Forest Nenets, respectively. Polymorphic variants frequencies for Nenets are intermediate between those in populations of Europeans and Asians. These results can probably be explained by the presence of both European and Asian components in Nenets gene pools.

**Conclusions:**

The results of this study expand the knowledge of *NAT2* polymorphism in world populations. These data may also help assess the genetic predisposition of Nenets to multifactorial diseases associated with polymorphism in the *NAT2* gene and, in general, contribute to the development of personalized medicine in reference to native people of Siberia.

## Background

A person’s life expectancy and quality of life are determined by many factors, including heredity, lifestyle, physical activity, nutrition, stress, and the exposure to foreign substances (xenobiotics). These include exotoxins, mutagens and carcinogens in tobacco smoke, food, air, medicines, industrial and agricultural products [1–3].

Foreign substances entering the body are metabolized by the enzymes of the xenobiotic biotransformation system (XBS). XBS genes are highly polymorphic. Numerous studies have shown that the combination of adverse environmental factors with the inheritance of unfavorable polymorphic variants of genes, which include XBS genes, determines individual sensitivity to various toxins. Such sensitivity may contribute to the development of socially significant diseases in both adults and children. Similarly, resistance or hypersensitivity of an individual to a particular drug may lead to lack of therapeutic effect or development of adverse reactions in response to treatment [4–14].

The process of xenobiotic metabolism in the body occurs in three stages or phases. The first phase, the activation phase, is carried out by a large family of cytochrome P450 enzymes (CYPs). The second phase involves various transferases and hydrolases that neutralize hydrophilic and often toxic phase I products. During the third phase, the xenobiotic compounds may are removed from cells by energy-dependent transporters to the extracellular medium, where they may be further metabolized or excreted [15, 16].

For the most effective neutralization of many foreign substances, it is necessary to have the combined action of enzymes from phases I and II. It has been revealed that the desynchronization of the biotransformation phases leads to the accumulation of peroxidation products, carcinogens and mutagens in the organism, leading to its rapid poisoning. Especially unfavorable is the combination of high activity of phase I enzymes and low activity of phase II enzymes [17–19]. Among the phase II enzymes, the families of glutathione-S-transferases (GSTs) and N-acetyltransferases (NATs) display the widest and most diverse activity [20].

There are two functional isoenzymes in NAT family, which metabolize a wide range of xenobiotics. These include carcinogens such as aromatic amines (4-aminobiphenyl), heterocyclic amines (2-amino-1-methyl-6-phenylimidazo [4,5-b]pyridines) contained in cigarette smoke and food [21]. The enzyme NAT1 is involved in the metabolism of folate, para-aminosalicylic and para-aminobenzoic acids. NAT2 is considered the main enzyme of xenobiotic acetylation. It is responsible for the biotransformation of a wide range of clinically important drugs (antihypertensive, hydrazine, hydralazine, phenelzine; arylamine drugs, including antiarrhythmic procainamide and sulfametazine of the antibacterial spectrum, anti-tuberculosis isoniazid, etc.) [22].

The *NAT1* and *NAT2* genes are located on the same chromosome, in the 8p21.3–23.1 region. These genes do not contain introns in their structure, have an open reading frame with a length of 870 nucleotide pairs, and have 87% of the nucleotide sequence homology of the coding region, but are regulated independently of each other [21, 23, 24]. *NAT1* is expressed in most body tissues, while *NAT2* is active in the liver, intestine, and breast tissue [21–23, 25, 26].

The *NAT1* and *NAT2* genes are highly polymorphic [21]. Our study is focused on the *NAT2* gene since it is considered the main enzyme of xenobiotic acetylation [22]. There are 16 polymorphic sites in this gene, including 15 SNPs and one deletion resulting in a frame shift [27]. Different combinations of these polymorphisms produce 36 variants of the *NAT2* gene; each of these combinations encodes an enzyme with different acetylation rate. In the *NAT2* gene, the “wild” type variant that is responsible for “fast” acetylation is referred to as NAT2*4. Three main “slow” variants have also been identified. The NAT2*5 variant (rs1801280, *T341C*) leads to the replacement of isoleucine with threonine at the position 114 of the protein molecule and causes a decrease in the maximum acetylation rate of N-acetyltransferase 2. Variant NAT2*6 (rs1799930, *G590A)* results in the replacement of arginine with glutamine at the position 197 of the protein molecule. And the third variant is the NAT2*7 (rs 799931, *G857A)* leading to the replacement of glycine with glutamine in the 286th position of the protein chain. The latter two variants cause the formation of a less stable enzyme molecule. Heterozygotes for the “fast” and “slow” variants display intermediate rate of acetylation [21, 28–30].

The frequencies of “slow” variants of the *NAT2* gene vary in human populations. It is well known that the average frequency of NAT2*5 is 50% among Europeans, 33–42% among Africans, and it is quite low (about 5%) among Asians. The NAT2*6 is common in all of these populations with a frequency of about 30%. As for the NAT2*7, its frequency is low enough for Europeans (less than 2%) and Africans (3–6%), and it reaches 10–12% among Asians [31–34]. It has been shown that NAT2*6 and NAT2*7 frequencies are 32.9 and 2.7%, respectively, in Europeans from Novosibirsk, Russia [35]. Among the Europeans of the Moscow region, the frequencies of NAT2*5, NAT2*6 and NAT2*7 variants were found to be 46.8, 19.3 and 2.9%, respectively [36].

Studying polymorphisms in the *NAT2* gene is clinically important due to the association identified between polymorphisms in this gene and the development of various socially significant diseases, as well as the sensitivity of individuals to drugs, such as isoniazid, that are used to treat tuberculosis [1, 37]. Numerous studies have shown a reliable link between polymorphic variants of *NAT* genes and the risk of cancer, including cancer of the head and neck, lungs, mammary glands, larynx, bladder, digestive tract [38–45]. The presence of a “slow” *NAT2* genotype in combination with a “zero” *GSTM1 (0/0)* genotype is a risk factor for the development of lymphatic leukemia in children [46]. *CYP1A1 Val*, NAT2*6 (*G590A*) variants and *GSTM1 (0/0)* genotype are associated with a predisposition to bronchial asthma development in children [47]. A study conducted in the Krasnodar Region of Russia showed that the presence of the NAT2*6 (590A/A) genotype increases by a factor of 3.5 the risk of developing congenital malformations such as cleft lip and/or palate in females compared to males [48].

Certain *NAT2* genotypes and some lifestyle features can be considered as combined risk factors for the development of psoriasis in a sample of Muscovites [49]. It has been shown that polymorphic variants of the *GSTT1*, *GSTM1*, and *NAT2* genes can potentially modulate the risk of tuberculosis development in ethnic Russians [50]. Certain polymorphic variants of *GSTT1*, *GSTM1*, *NAT2*, and *MTRR* genes can modulate the risk of acute leukemia in children living in the European part of Russia [51]. In Yakuts, the allele NAT2*7 (857A) and the genotype NAT2*7 (857 G/A) are markers of increased risk of lung cancer [52].

Since the 1960s, in the northern areas of Western Siberia, the development of industry has been growing. This had led to changes in the traditional way of life for indigenous ethnic groups and the introduction of new chemicals, drugs and toxic pollutants into their habitats. Therefore, it is necessary to determine whether these indigenous ethnic groups are resistant to these xenobiotics and whether there is a high risk of exposure of the population that leads to serious health issues, such as respiratory diseases, cancer and allergies. For this purpose, it is necessary to study the genetic profile of indigenous populations and their ability to metabolize xenobiotics.

Ethnography, anthropology, demographic history of indigenous Nenets populations and genetic markers such as polymorphism in genes *GSTM1*, *GSTT1*, *CYP2C9*, and *CYP2D6* associated with xenobiotics metabolism have been studied previously [53–61].

In this study, we analyzed the occurrence of NAT2*5 (rs1801280, *T341C)* and NAT2*7 (rs1799931, *G857A)* variants of the *NAT2* gene in the populations of Tundra and Forest Nenets of the Yamalo-Nenets Autonomous Okrug (YaNAO).

## Results

The frequencies of the studied polymorphic variants of the *NAT2* gene in Nenets and other world populations are shown in Table 1. The data were collected according the established protocols [67] and the results were confirmed by the statistical estimates [68, 69].

**Table 1:** NAT2*5 and NAT2*7 variant frequencies of the *NAT2* gene in some human populations, %

Population	NAT2*5 (341C)	NAT2*7 (857A)
Tundra Nenets^a^, YaNAO, Russia	28.0 (*n* = 182)	9.8 (*n* = 194)
Forest Nenets^a^, YaNAO, Russia	38.6 (*n* = 290)	8.2 (*n* = 330)
Tatars, Bashkiria region, Russia [62]	No data	9.5 (*n* = 136)
Europeans, Moscow, Russia [49]	42.0 (*n* = 198)	3.0 (*n* = 198)
Russians, Voronezh region, Russia [63]	42.0 (*n* = 580)	3.0 (*n* = 580)
Germans, Germany [31]	46.5 (*n* = 1688)	1.3 (*n* = 1688)
Romanians, Central Europe [64]	43.5 (*n* = 280)	3.2 (*n* = 280)
Egyptians [65]	49.7 (*n* = 400)	2.8 (*n* = 400)
Kyrgyz, Central Asia [64]	19.3 (*n* = 580)	12.1 (*n* = 580)
Han Chinese, China [66]	1.8 (*n* = 224)	17.0 (*n* = 224)
Bantu, Africa [34]	38.3 (*n* = 204)	6.1 (*n* = 204)

*n* number of alleles examined; ^a^own study

### NAT2*5 T341C (Ile114Thr)

Genotype counts in Forest and Tundra Nenets were as follows: *TT* – 55 and 45; *TC* – 68 and 41; *CC* – 22 and 5 respectively. In each sample, the distribution of NAT2*5 (T341C) frequencies reflected Hardy-Weinberg equilibrium (χ^2^ = 0.017, *p* = 0.896 and χ^2^ = 1.244, *p* = 0.265 for Forest and Tundra Nenets).

The frequency of the “slow” NAT2*5 (T341C) variant in the Tundra Nenets was statistically significantly lower (28.0%) than in the Forest Nenets (38.6%), *p* = 0.024.

### NAT2*7 G857A (Gly286Gln)

Genotype counts in samples of Forest and Tundra Nenets were as follows: *GG* – 139 and 78; *GA* – 25 and 19; *AA* – 1 and 0, respectively. An additional file contains more detailed information on individual genotyping results (See Additional File 1). In both samples, the distribution of frequencies NAT2*7 (G857A) reflected Hardy-Weinberg equilibrium (χ^2^ = 0.012, *p* = 0.914 and χ^2^ = 1.143, *p* = 0.285 for Forest and Tundra Nenets, respectively).

The frequencies of the “slow” NAT2*7 (857A) variant in the study populations did not statistically differ from each other, with Forest and Tundra Nenets having essentially the same values (8.2 and 9.8%, χ^2^ = 0.22; *p* = 0.64).

## Discussion

As described above, the polymorphic variant NAT2*5 (341C) is found in the world populations with an average frequency of 30–50% among Europeans, 33–42% among Africans, and quite rarely among Asians (up to 5%). The results of our study showed that the frequencies of the NAT2*5 (341C) variant in Forest Nenets and Europeans did not statistically significantly differ. Tundra Nenets, by contrast, have a statistically significantly lower frequency of NAT2*5 compared to the comparative populations (Russians, Romanians, Germans, Egyptians, Bantu Africans), as well as Forest Nenets (in all cases of pairwise comparison *p* < 0.05). In addition, there are statistically significant differences between the Nenets and Kyrgyz, as well as with Han Chinese, for whom the frequency of NAT2*5 (341C) is low enough, as in other Asian populations (*p* < 0.05).

We have mentioned above that the frequency of NAT2*7 (857A) is low for Europeans (less than 2%) and Africans (3–6%), and among Asians it reaches 10–12%. Our study showed that with respect to the frequency of the NAT2*7 (857A) variant, both Forest and Tundra Nenets are statistically significantly different from Russians, Romanians, Germans, and Egyptians (*p* < 0.05), although there are no statistically significant differences with Africans, Kyrgyz, and Tatars.

In Forest and Tundra Nenets, the frequencies of “slow” polymorphic variants NAT2*5 and NAT2*7 of one of the key genes of the xenobiotics biotransformation system, including carcinogens and the main clinically important drugs, occupy an intermediate value between the frequencies among the considered populations of Europeans and Asians. Such results can probably be explained by the presence in the gene pools of the Nenets, both European and Asian components, which were determined according to anthropological and genetic data [53–60].

Based on our data on lower frequencies of the “slow” NAT2*5 variant in Nenets, compared with Europeans, we can assume that at the population level these northern peoples, unlike the studied European populations, are at a lower risk of developing cancer and other multifactorial diseases with which a polymorphic variant of NAT2*5 is associated. Especially since the standardized mortality rate from cancer in the Yamalo-Nenets okrug in 2017 fell below the level in the Russian Federation [61]. However, to confirm our assumption, it is necessary in the future to use additional population genetic parameters and the necessary statistical methods of data processing.

## Conclusion

Currently, changes in the habitual way of life of indigenous ethnic groups are taking place due to the crossbreeding of the population and the rapid development of industry in the Yamalo-Nenets Autonomous Okrug. New chemical substances and carcinogens penetrate into the Nenets’ habitat. The development of medicine, in turn, contributes to the active penetration of drugs. Changing standards of treatment require the use of more and more new medicines, which indigenous peoples have never encountered before.

Therefore, having an idea about polymorphic variants frequencies of the genes of the xenobiotic biotransformation system, which include medicinal products, and hence the peculiarity of their metabolism in indigenous Samoyedic ethnic groups, it is possible to plan the safest and most effective therapy with medicines. Knowledge about the distribution of polymorphic variants of genes in the xenobiotic biotransformation system allows us to identify genotypes associated with socially significant, multifactorial diseases, which makes it possible to identify predisposition to them and to take preventive measures to avoid the development of pathological conditions.

## Methods

### Study populations

To conduct the study, samples of representatives of Tundra and Forest Nenets living in the Purovsky region of the YaNAO were obtained. These samples did not include descendants of intermarriages with Russians and other ethnic groups. The material for the study was collected during the 1988–2009 expeditions to the YaNAO under the guidance of L. P. Osipova. Peripheral blood collection was carried out according to international rules [67] with the use of genealogical information and “Informed consent” from volunteers who were healthy at the time of the study.

### DNA isolation and SNP genotyping

DNA samples were isolated from the leukocyte fractions of venous blood by the standard method of phenol-chloroform extraction using proteinase K. Genotyping of NAT2*5 (*T341C*) and NAT2*7 (*G857A*) SNPs of *NAT2* gene was performed using real-time PCR with competing TaqMan-probes carrying a quencher at the 3′-end and different fluorescent dyes (FAM or R6G) at the 5′-end. Probe sequences for NAT2*5 (T341C) were as follows: 5′-FAM-CAGGTGACCATTGACGGCAG-BHQ-3′ и 5′-R6G-CAGGTGACCACTGACGGCAG -BHQ-3′.

Probe structures for NAT2*7 (G857A): 5′-FAM-CCAAACCTGGTGATGAATC-BHQ-3′ and 5′-R6G-CCAAACCTGGTGATGGAT-BHQ-3′. The primer sequences for NAT2*5 *(T341C) *were as follows:

- NAT2-RT-U1 agcactggcatggttcac
- *NAT2-RT-R3* GTTTCTTCTTTGGCAGGAGATGAG

In the case of NAT2*7 (G857A) primers had such structures:

- NAT2-RT-U3 ctgaggaagaggttgaagaagtg
- NAT2-803R GTTGGGTGATACATACAGAAGGG

The total volume of the reaction mixture was 8 μl, the mixture contained DNA with a concentration of 15 ng/μl, 300 nM of each primer; 100–200 nM each of Taqman probes conjugated with FAM or R6G; 200 μM dNTPs, amplification buffer (650 mM Tris-HCl, 240 mM (NH_4_)_2_SO_4_, 0.5% Tween 20, 35 mM MgCl_2_), thermostable Taq polymerase - 0.5 units.act./react. PCR was performed under the following conditions: initial denaturation 5 min at 95 °C; then 48 cycles including denaturation at 95 °C for 10 s, annealing of primers and subsequent elongation at 60 °C for 40 s (each step was accompanied by registration of the fluorescence signal in the ranges corresponding to the fluorescence intervals of FAM and R6G fluorophores). The work was carried out using the amplifier Bio-Rad CFX manager (USA). The obtained data were processed with the help of “CFX manager” software.

### Statistical analysis

The population frequencies of polymorphic variants were calculated on the basis of the observed genotype frequencies. The conformity of genotype frequencies to the Hardy-Weinberg equilibrium was assessed using the χ^2^ (Pearson) criterion, using the on-line test program (at *p* > 0.05 the equilibrium is performed) [68]. In order to assess the reliability of the differences in the frequencies of polymorphic variants between population samples, the χ^2^ criterion with Yates correction was applied (at *p* < 0.05, the results were considered to be reliably significant) [69].

## Supplementary information

**Additional file 1. **Results of genotyping of point substitutions in *NAT2* gene. File containing the results of genotyping (individuals genotypes) of point substitutions NAT2 * 5 T341C (rs 1801280) and NAT2 * 7 G857A (rs 1799931) by Real-time PCR. The study involved healthy volunteers, representatives of indigenous peoples, Forest Nenets and Tundra Nenets living in Purovsky district of Yamalo-Nenets Autonomous Okrug, Russian Federation, settlement Samburg for the Tundra Nenets and settlements Kharampur, Khalyasavey, Tarko-Sale for the Forest Nenets. All research participants gave a written informed consent to the clinical examination and the publication of their anonymous data.

## Data Availability

All data obtained and analyzed during this study is included in this published article. Individual genotyping data are available at the Novosibirsk State University site (http://lcg.nsu.ru/en/links/). The data obtained for each of the studied genetic samples are also provided in an Additional file 1.

## Abbreviations

DNA: Deoxyribonucleic acid
dNTPs: deoxynucleotide triphosphates
NAT: N-acetyltransferase
PCR: Polymerase chain reaction
YaNAO: Yamalo-Nenets Autonomous Okrug
XBS: Xenobiotic biotransformation system
